# Plasmonic field-regulating characteristics of alloy-based multilaminar films in 300–800 nm

**DOI:** 10.1016/j.heliyon.2023.e13084

**Published:** 2023-01-20

**Authors:** Yifan Kang, Hongtao Yang, Chao Wang, Yongfeng Li, Peng Xu

**Affiliations:** a Xi'an Institute of Optics and Precision Mechanics of Chinese Academy of Sciences, Xi'an 710119, China; b University of Chinese Academy of Sciences, Beijing 100049, China; c Air Force Engineering University, Xi'an 710051, China; d Center for Attosecond Science and Technology, State Key Laboratory of Transient Optics and Photonics, Xi'an Institute of Optics and Precision Mechanics of Chinese Academy of Sciences, Xi'an 710119, China; e Department of Microelectronics, School of Electronic Engineering, Xi'an University of Posts & Telecommunications, Xi'an 710121, China

**Keywords:** Multilaminar metamaterial composites, Field-regulating characteristics, Alloy plasmonic effect, Absorption saturation effect, Magnetic resonance

## Abstract

Based on a petal-like microstructure model of alloy particles we proposed, the field-regulating characteristics of alloy-based metamaterial films in the wavelength range of 300–800 nm are analyzed. It is found that Au/Ag alloy particles can support a broader resonance band with higher averaged resonance intensities than that of pure silver or gold particles, which, named alloy plasmonic effect, proves to be a universal feature of alloy-based plasmonics. Upon optimizing the coupling interaction between the alloy plasmonic effect and absorption saturation effect within alloy-based multilaminar structures, a broadband electromagnetic wave absorber consisting of a Cu/Al alloy-based composites layer and an aluminum base layer is demonstrated. Furthermore, a generalized method is proposed to evaluate the absorption performance of this kind of plasmonic absorber. The achieved alloy-based absorber proves to be nearly non-iridescent and the quality factor AP throughout the range of 300–800 nm remains higher than 0.8 even if the incident angle increases up to 60°.

## 1 Introduction

The interaction between the electromagnetic radiation and matter is the footing stone of all optical physics [1]. Reflection, absorption and transmission are not only important macroscopic parameters for describing the field-regulating characteristics of those physical processes, but also the main themes of typical applications as antireflective coatings [2,3,4,5,6,7,8] and perfect absorber [9,10,11,12,13,14,15,16]. For the time being, the emerging optoelectronic applications are placing urgent demands on functional materials with specified properties: broadband, ultrathin thickness, flexibility, non-iridescent. For example, the infrared camouflage requires the materials with selective emissivity throughout the range of 3–14 μm. In essence, they are calling for materials tailored with novel properties beyond the natural media. The emergence of metamaterials meets these requirements and provides even new opportunities for these applications [17,18,19,20]. Metamaterials are artificial materials for realizing the expected spectral response behaviors by the interactive plasmonics behaviors of unit structures. Since experimentally demonstrated in 2001 [21], they have realized novel unnatural properties and resulted in great performance improvement of related devices [22,23]. Until now, metamaterials-based applications have made great progress, such as the broadband perfect absorber in the visible, antireflective metasurfaces, and transparent metal film.

As for metamaterials-based functional materials, the main physical processes involved are either or both of surface plasmon polaritons (SPPs) and localized surface plasmons (LSPs). Based on our findings [24], LSPs is the main physical process to determine the interaction of electromagnetic radiation with plasmonic structures. To achieve broadband performance, more LSPs at different frequencies must be excited within the materials. So it is necessary to introduce two or more kinds of metal nanoparticles into the composites to broaden the plasmonic resonance range. In recent years, there have been a few reports about alloy-based composite films [25,26,27]. Nevertheless, no such research has been reported about the generalized method to analyze the plasmonic characteristics of alloy-based composite films. And it is not clear yet about how the compositional proportion of each metal component influences the resonance process and eventually determines the broadband performance of materials. This hinders the development of alloy-based functional materials with desirable field-regulating properties.

The main purpose of this text is to elaborate the generalized method of analyzing the field-regulating characteristics of alloy-based composites. Firstly, a microstructure model is established to simulate the plasmonic characteristics of alloy particles and the plasmonic field-regulating properties of alloy-particle-based composites are analyzed. It is found that the alloy particles support a broader resonance band with higher averaged resonance intensities than that of pure metal particles. Secondly, multilaminar composites composed of an alloy-particle-based composites layer and a base metal layer are further explored. Upon optimizing the coupling interaction between the alloy plasmonic effect and absorption saturation effect, a non-iridescent broadband absorber throughout the range of 300–800 nm is demonstrated. The absorption performance is characterized by the evaluation method proposed.

## 2 Results and discussions

### 2.1 Field-regulating characteristics of alloy-particles-based composites

**Fig. 1:**
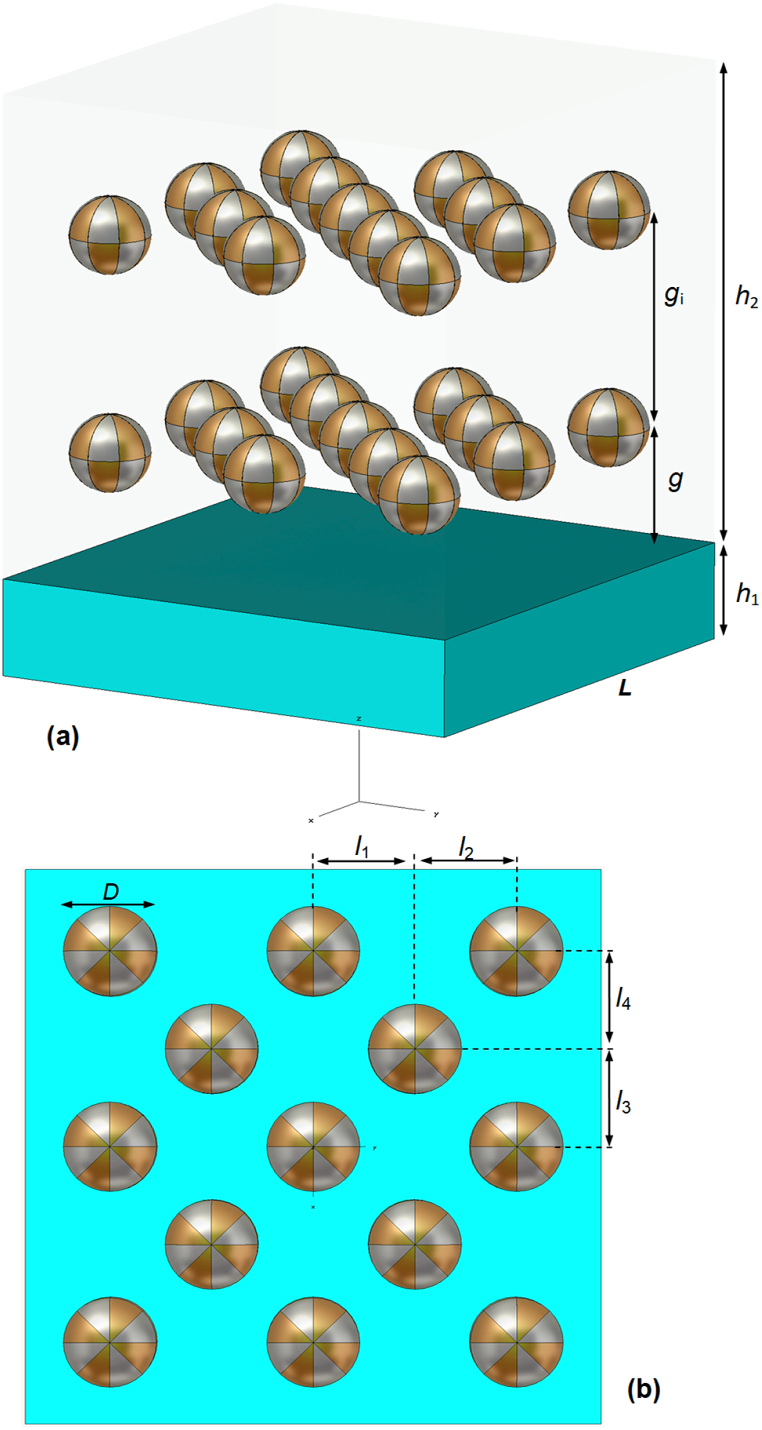
Schematics of the unit cell of multilaminar metamaterial structure. (a) Isometric view; (b) vertical view.

The schematics of typical multilaminar metamaterial films for analysis are illustrated in Fig. 1(a), in which all the structural parameters are illustrated. The bottom layer is a base metal layer of thickness $h_{1}$ and the upper one is an alloy-particles-based dielectric composites layer of thickness $h_{2}$. All alloy particles are assumed to be spherical with diameter D, though they can be in any other shapes. For the sake of simplicity, we also assume that the alloy particles are regularly distributed in layers in dielectric matrix, as shown in Fig. 1(b). The parameter g refers to the distance in the Z direction between the lower (inner) layer of alloy particles and the upper surface of metal film, and $g_{i}$ the interval between these two sublayers of alloy particles. The transverse positions of alloy particles within the unit cell are defined by four parameters $l_{1}$, $l_{2}$, $l_{3}$, and $l_{4}$ as illustrated. The excitation source propagating along the negative Z axis is a TE-polarized plane wave with polarization direction along the Y axis. The normal incidence is considered, unless otherwise stated. To mimic the practical situation of applications, periodic boundary conditions are set for both the X and Y boundaries of unit cell. The side length of unit cell is fixed L=100 nm, and the frequency spectrum range of interest is 375–1000 THz, i.e., 300–800 nm in wavelength. Polytetrafluoroethylene (PTFE) is used as matrix for all cases. The permittivity constant of PTFE is 2.1 and loss tangent is 0.0002 in this frequency region. To guarantee the correctness of simulation, we first use CST Microwave Studio and Optiwave OptiFDTD for comparison analysis and finally adopted the former for all the following analysis. From the simulation, one can obtain the two intermediate parameters, $S_{11}$ and $S_{21}$, and further deduces the overall field scattering characteristics, the reflection $R={\left|S_{11}\right|}^{2}$, the transmission $T={\left|S_{21}\right|}^{2}$, and the absorption A=1−R−T. In the following analysis, four types of metals are involved, gold (Au), silver (Ag), copper (Cu), and aluminum (Al). The dielectric permittivity of each metal is from the data by Palik [28].

**Fig. 2:**
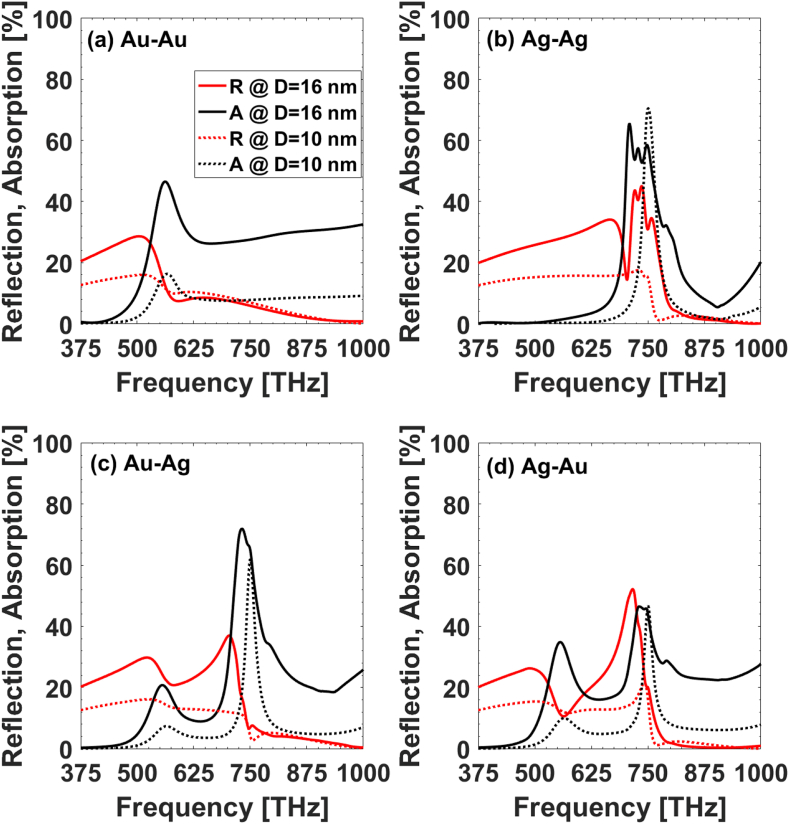
Plasmonic characteristics of composites film embedded with both silver and gold nanoparticles. (a) Au–Au; (b) Ag–Ag; (c) Au–Ag; and (d) Ag–Au combination. (For interpretation of the references to colour in this figure legend, the reader is referred to the Web version of this article.)

Firstly, we consider the simplified structure case with $h_{1}=0$. Other parameters are $g_{i}=25$ nm, $l_{1}=l_{2}=l_{3}=l_{4}=17.5$ nm. To differentiate from the real alloy structure mentioned in next paragraphs, when each particle embedded is made of pure metal, the structure architecture is named metal combination. The combination metal_1–metal_2 means that the metal particles in outer and inner sublayers are made of pure metal_1 and pure metal_2, respectively. In visible frequency range, i.e., 300–800 nm, since gold and silver are two commonly used metals in plasmonics, we choose them as example metals to do the analysis. For the structure embedding two sublayers of particles, the field-regulating characteristics are given in Fig. 2. For the two combinations of Au–Au and Ag–Ag with D=10 nm in Fig. 2(a) and (b), the resonance peaks appear at 565 THz and 750 THz, respectively. This is the intrinsic plasmonic resonance of each case and the resonance bandwidth is narrow. When the particle size is getting large, e.g., D=16 nm, the volume filling of metal in matrix is becoming higher and the coupling interactions between metal-particle-centered plasmons get stronger. Although there is observable change of resonance bandwidth, the center frequency is still located around at the intrinsic resonance frequency of gold and silver particles, respectively. While for the combinations of Au–Ag and Ag–Au in Fig. 2(c) and (d), the resonance bandwidth is broadened apparently. When the volume filling of metal in matrix is high, the interactions between metal-particle-centered plasmons are more complicated and result in more frequency redshifts and blueshifts. On the other side, it is noteworthy that the field-regulating characteristics of Au–Ag and Ag–Au combinations differ greatly. Compared with Au–Au, although both Au–Ag and Ag–Au combinations have suppressed the resonance at around 565 THz and simultaneously enhanced the resonance at around 750 THz, Au–Ag combination has more pronounced change. If compared with Ag–Ag, the results are different and Ag–Au has more significant changes. Even without detailed analysis, one can easily know that it originates from not only the difference of the spatial distributions of metal particles in matrix, but also the difference of plasmonic properties between silver and gold particles. This field-regulating property is dependent on the incidence direction of excitation and should be eliminated in practical applications.

**Fig. 3:**
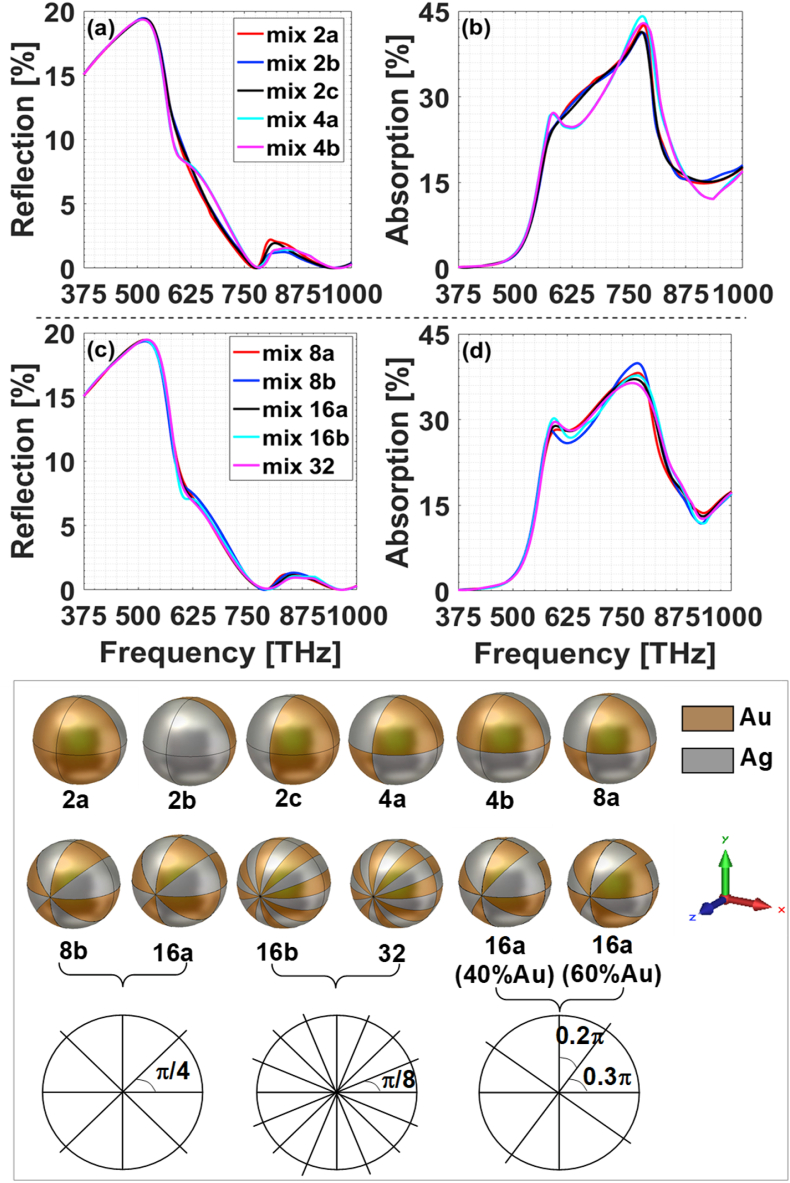
Plasmonic characteristics of one-sublayer-alloy-based composites film. The volume filling ratio between silver and gold components in each particle is 1:1. (a) Reflection, and (b) Absorption for structures with fewer petals; (c) Reflection, and (d) Absorption for structures with more petals. The bottom panel illustrates the microstructure of the alloy particle. (For interpretation of the references to colour in this figure legend, the reader is referred to the Web version of this article.)

To achieve the performance of incidence-direction-independent broadband response, alloy-particle-based composite films proved to be an effective resort [25]. For designing such films, one of the unavoidable issues therein is how to describe the microstructure of the alloy particle and to establish an appropriate model for analysis. For a real alloy particle, various metal components are uniformly dispersed therein in the form of microspheres. In this sense, the issue of modeling comes down to determine the subdivision degree of each alloy particle. As a start, Au/Ag alloy particle is considered and the volume ratio between two components is 1:1. For the composites embedding with one sublayer of Au/Ag alloy particles with D=10 nm, the results for different subdivided microstructures are given in Fig. 3. With the synchronous increasing of both metal petals, the two components are mixed more evenly and the microstructure gets closer to reproduce the true state of practical material synthesis such as magnetron sputtering. When the number of petals in each alloy particle reaches sixteen or even more, both the field reflection and absorption by the alloy-based composites are beginning to stabilize in the whole frequency band, as shown in Fig. 3(c) and (d). So one can safely argue that the sixteen-petal structures have been subdivided enough to approximate the microstructure of real alloy particles.

**Fig. 4:**
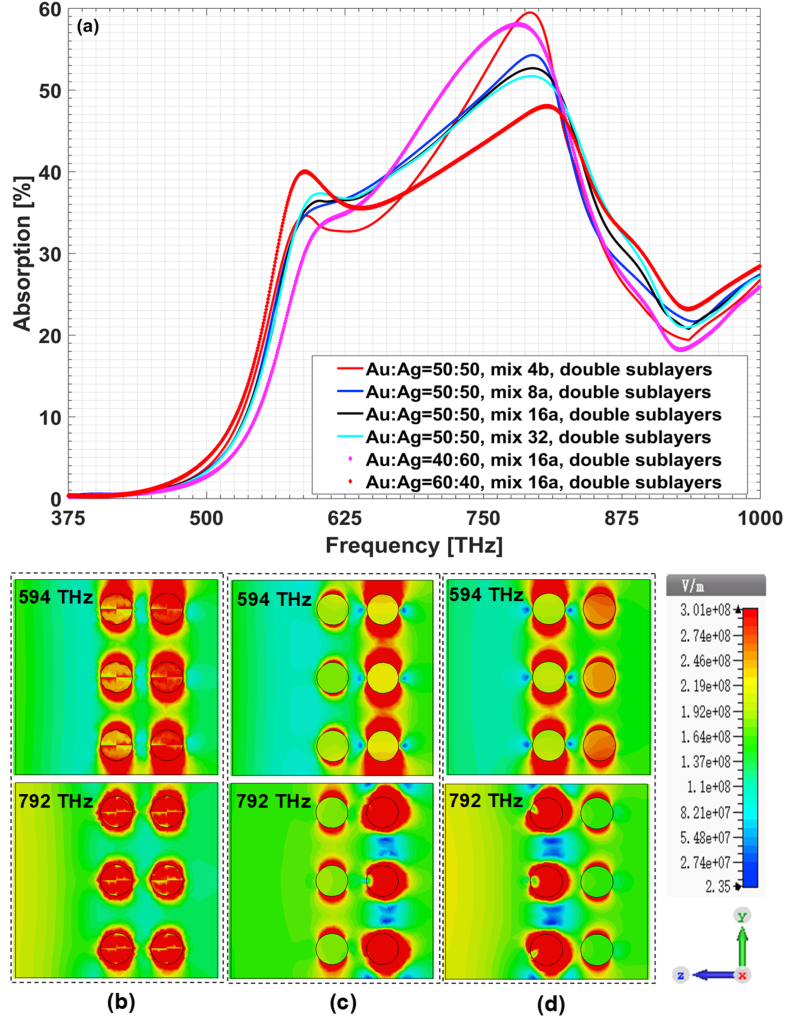
Plasmonic characteristics of double-sublayer-alloy-based composites film. (a) Absorption spectrum; electric field distribution in x=0 longitudinal section for (b) alloy case (mix 16a), Au:Ag = 50:50; (c) Au–Ag and (d) Ag–Au combination.

**Fig. 5:**
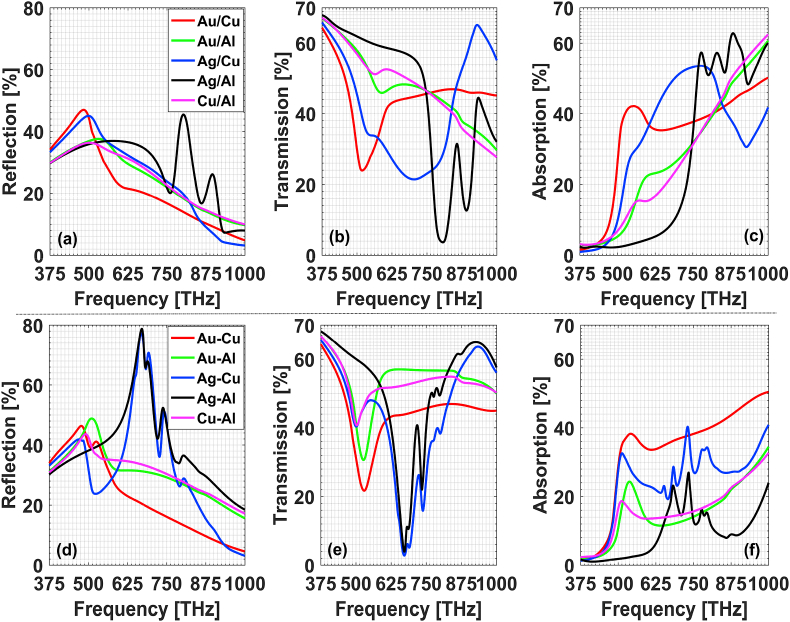
Plasmonic characteristics of different structure architectures: (a) reflection, (b) transmission, (c) absorption for metal alloys; (d) reflection, (e) transmission, (f) absorption for metal combinations.

To confirm this assertation, we further analyzed the structures embedding with double sublayers of Au/Ag alloy particles as in Fig. 4. Here $g_{i}=25$ nm. One can know that the field-regulating characteristics of sixteen-petal subdivided microstructure are the convergence of these comparison cases with increased subdivision petals. Compared with the field-regulating properties of Au–Ag and Ag–Au combinations shown in the lower panels of Fig. 2, Au/Ag alloy-particle-based composites possess weakened reflection and enhanced absorption in the whole frequency band. To get more details about the underlying physical mechanism, we also plot the electric field distributions in the longitudinal section of x=0. It shows that for Au–Ag or Ag–Au combination in Fig. 4(c) and (d), the resonances at 594 THz and 792 THz are mainly located around the silver particles. While for the Au/Ag alloy-based composites in Fig. 4(b), each alloy particle contributes almost equally to both frequency resonances and the alloy-particles-based composites have stronger plasmonic resonances and stronger averaged absorption. This can be called *alloy plasmonic effect*. We have tried other type of alloy particles and displayed the field-regulating characteristics in Fig. 5(a)–(c). For comparison, the results for the corresponding metal combinations are also given in Fig. 5(d)–(f). One can draw the conclusion that alloy plasmonic effect is a universal feature of alloy-based plasmonics. For other alloy particles with different proportions of metal components, it can also be dealt with the method just illustrated. As an exemplification, we also give in Fig. 3 the subdivided microstructure of alloy particle with 40 or 60 percents of gold component and in Fig. 4 the calculated absorption characteristics. One can see that the volume filling ratio between different metal components can serve as an important handle for further tailoring the field-regulating characteristics of alloy-particle-based composites.

**Fig. 6:**
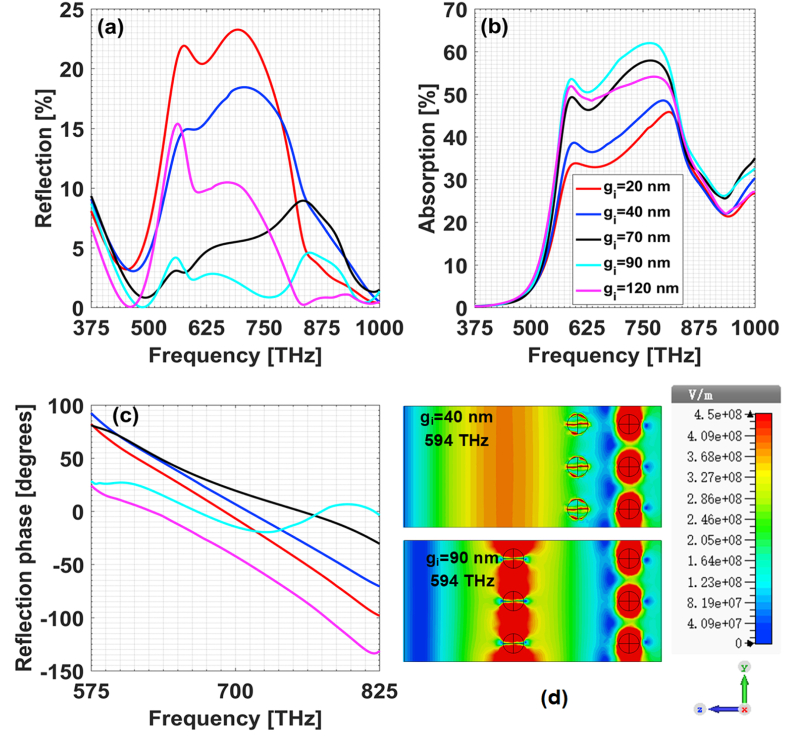
Dependence of field-regulating characteristics on $g_{i}$. (a) Reflection spectrum; (b) absorption spectrum; (c) reflection phase shifts; (d) electric field distribution in x=0 longitudinal section.

As for the influence of other structural parameters, we give in Fig. 6 the dependence of both reflection and absorption spectrum on $g_{i}$. The particles diameter is D=10 nm and the volume ratio between gold and silver component in each alloy particle is still 1:1. When the two sublayers of Au/Ag alloy particles are close, e.g., $g_{i}=20$ nm, the reflection is high and the absorption therein is low. Along with the increasing of $g_{i}$, the changes of reflection and absorption in resonance band 575–800 THz are not monotonous, as shown in Fig. 6(a) and (b). The structure with $g_{i}=90$ nm possesses the weakest reflection and strongest absorption. To get more details about the physical mechanism, we plot in panel Fig. 6(d) the distributions of electric field in the longitudinal section of x=0 for $g_{i}=90$ nm and $g_{i}=40$ nm. The reflection phase shifts are also calculated and shown in Fig. 6(c). One can know that the reflection phase shifts for $g_{i}=90$ nm are around zero, while the reflection phase shifts for $g_{i}=40$ nm are otherwise much far away from zero. We infer that the underlying physical mechanism is wave interference. When the incoming excitation field is incident on the composites, it can be reflected directly by the outer sublayer of alloy particles, forming the primary reflected electromagnetic field. The transmitted field can undergo multiple reflections between the inner and outer sublsyers of alloy particles and transmit back through the outer alloy sublayer, forming the secondary reflected electromagnetic field. The interference effect of all the reflected electromagnetic fields will determine the field-regulating characteristics of the composites. For $g_{i}=90$ nm, the two sublayers of alloy particles are acting as the resonant centers just as two cavity faces of a laser resonator. So the field power is greatly confined between the alloy particles and the field reflection reaches the minimum. The reflected electromagnetic fields are just like the output of a laser resonator and the phase curves are relatively flat. While for $g_{i}=40$ nm, the first sublayer of alloy particles is not providing decent contributions to plasmonic resonances as that for $g_{i}=90$ nm. So the reflection is high and much less power is confined within the composites. From this, it can be further deduced that if the volume ratio between different metal components in each alloy particle are changed, the value of $g_{i}$ needs to be otherwise optimized for maximum absorption.

### 2.2 Alloy-based multilaminar composite films

**Fig. 7:**
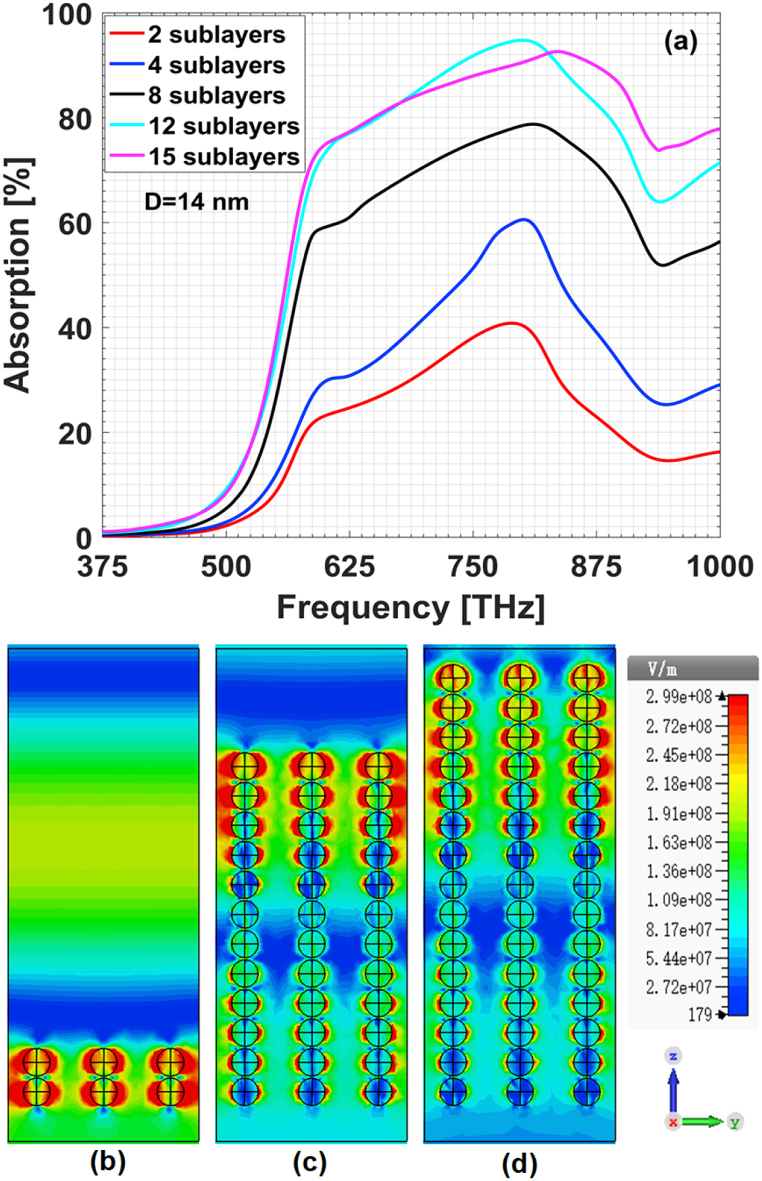
Absorption saturation effect of alloy-particles-based composites: (a) absorption spectrum; electric field distribution at 600 THz in the longitudinal section of for (b) two, (c) twelve, (d) fifteen sublayers.

In this section, our appeal is to achieve high efficient broadband absorption throughout 375–1000 THz. First of all, we consider the absorber based on multilayers of Au/Ag alloy particles. The volume ratio between gold and silver components in each Au/Ag alloy particle is still assumed to be 1:1. Other parameters are $h_{2}=250$ nm and $g_{i}=15$ nm. The field-regulating characteristics are given in Fig. 7(a). With the increasing of the number of alloy sublayers, one can see that the absorption spectrum does not increase monotonously but shows a saturation trend. This means that embedding more alloy particles within a given dielectric matrix does not definitely enhance the absorption. This phenomenon is called absorption saturation effect. To dig the underlying physical mechanism, we plot the electric field distribution at 600 THz in the longitudinal section of x=0 for three cases as shown. When there are two sublayers, each alloy particle contributes almost equally to the plasmonic resonance as shown in Fig. 7(b). While for such cases with twelve or even fifteen sublayers, some alloy particles make much less contributions to the resonance and thus the field confinement within the matrix arrives at saturation, as shown in Fig. 7(c) and (d). In brief, it is not feasible to achieve broadband electromagnetic wave absorber only by increasing the volume filling of alloy particles within the matrix.

**Fig. 8:**
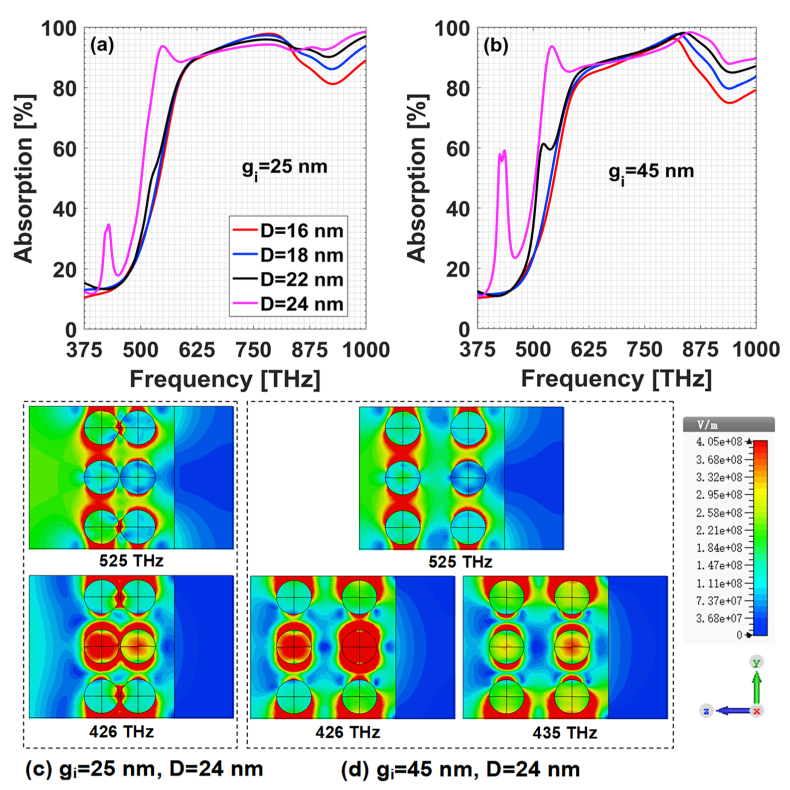
Absorption spectrum of composites PTFE:Au/Ag + Au. Absorption for (a) $g_{i}=25$ nm, (b) $g_{i}=45$ nm; electric field distribution in longitudinal section of x=0 for (c) $g_{i}=25$ nm, D=24 nm; (d) $g_{i}=45$ nm, D=24 nm.

As for the general multilaminar composite films with a base layer, the structure parameters are set as $h_{1}=40$ nm, $h_{2}=100$ nm, and g=25 nm. The base layer is gold. The volume ratio between Au and Ag components in each Au/Ag particle is 1:1. For the sake of convenience, the composites are narrated by PTFE:Au/Ag + Au. The dependence of absorption characteristics on particles parameters is simulated and displayed in Fig. 8(a) and Fig. 8(b). It shows that when the particle size is small, e.g., D=18 nm, the coupling between alloy-particle-centered plasmonic resonances is weak and both parameters of $g_{i}$ and D have little influence on the absorption spectrum. Along with the increasing of alloy particles size, the plasmonic coupling interactions become stronger and more resonances peaks appear in the low frequency range. As can be seen from the electric field distributions in Fig. 8(c) and (d), the absorption peak at 525 THz is originated mainly from the coupling interactions within each alloy sublayer (called intra-sublayer coupling). Therefore, $g_{i}$ has little impact on the absorption at 525 THz. While for the absorption at 426 THz, the coupling interactions between two alloy sublayers (called inter-sublayer coupling) play a comparable role in forming the plasmonic resonance. When these two alloy sublayers get too closer as $g_{i}=25$ nm, the inter-sublayer coupling is strong so that it suppresses the overall resonant effect by greatly weakening the intra-sublayer coupling. The direct result is that the two absorption peaks at 426 THz and 435 THz when $g_{i}=45$ nm merge into one at around 426 THz when $g_{i}=25$ nm. In summary, it is necessary to comprehensively tailor the interaction both the inter- and intra-sublayer coupling to achieve broadband absorption spectrum.

**Fig. 9:**
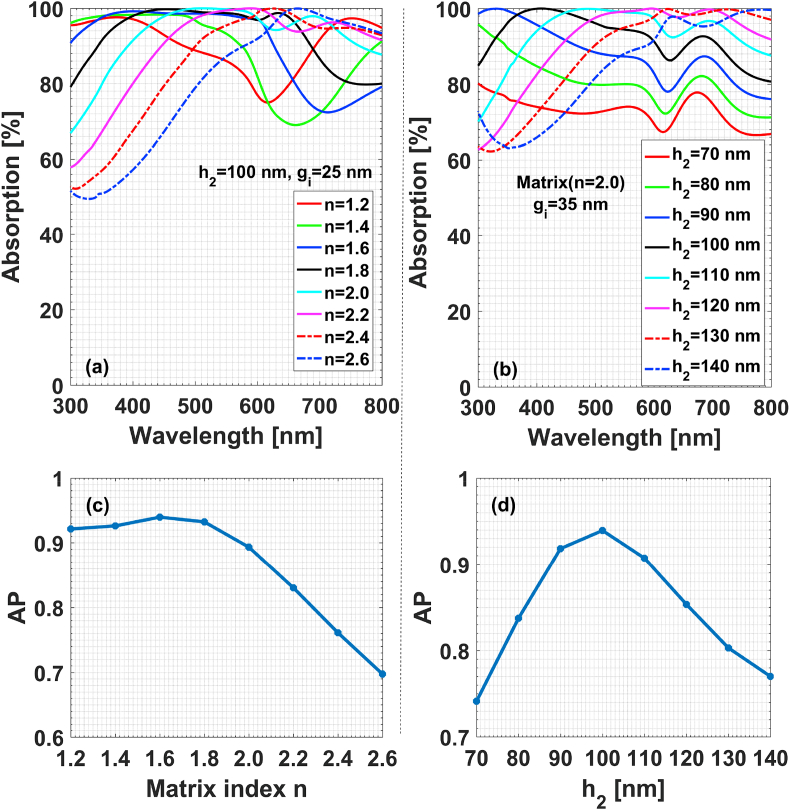
Effect of structure parameters on the absorption performance of composites Matrix(n):Cu/Al + Al. (a) Absorption, and (c) AP factor changing with matrix index; (b) absorption, and (d) AP factor changing with matrix thickness.

Based on the previous study [24], metals can be qualitatively classified into long- and short-range plasmonic ones according to the evanescent decay length of localized plasmonic fields in dielectric. For the four metals mentioned in this following text, the order of the evanescent decay length of localized plasmonic fields in 375–1000 THz is approximately Ag > Au (or Cu) > Al. Compared with the composites structure detailed in previous section, the objective of additionally introducing a base metal layer is to reflect the electromagnetic field transmitted through the alloy-particle sublayers back and hence to enhance the localized plasmonic interaction therein. Taking all these factors into consideration, the metal layer is chosen to be Al film with thickness $h_{1}=40$ nm and the particle is Cu/Al alloy. Cu instead of Au is adopted to lower the material preparation cost. To increase the contributions to the field confinement from each alloy sublayer, D=24 nm and g=15 nm are purposely set to approach the percolation of matrix. The absorption characteristics are given in Fig. 9, in which n refers to the matrix refractive index. To make a quantitative comparison between these structure cases, we purposely introduce a quality factor, absorption performance (AP), to quantify the absorber absorption efficiency of external electromagnetic field. AP is defined by the ratio between the absorbed power and the incident power. Fig. 9 shows that the structural parameters have significant impact on absorption performance of multilaminar composites. Both Fig. 9(a) and (c) show that the matrix with low refractive index will help enhance the absorption in high frequency range, while the matrix with high refractive index favors the absorption in low frequency range. Meanwhile, it is worth noting that the thickness of matrix has such remarkable influences that AP can drop from 0.94 to 0.74 when $h_{2}$ merely has a change of 30 nm from its optimal value of $h_{2}=100$ nm, as shown in both Fig. 9(b) and (d). When changing the volume ratio between Cu and Al components, one can easily infer that absorption performance can be further optimized to get broadband perfect absorber in 300–800 nm.

**Fig. 10:**
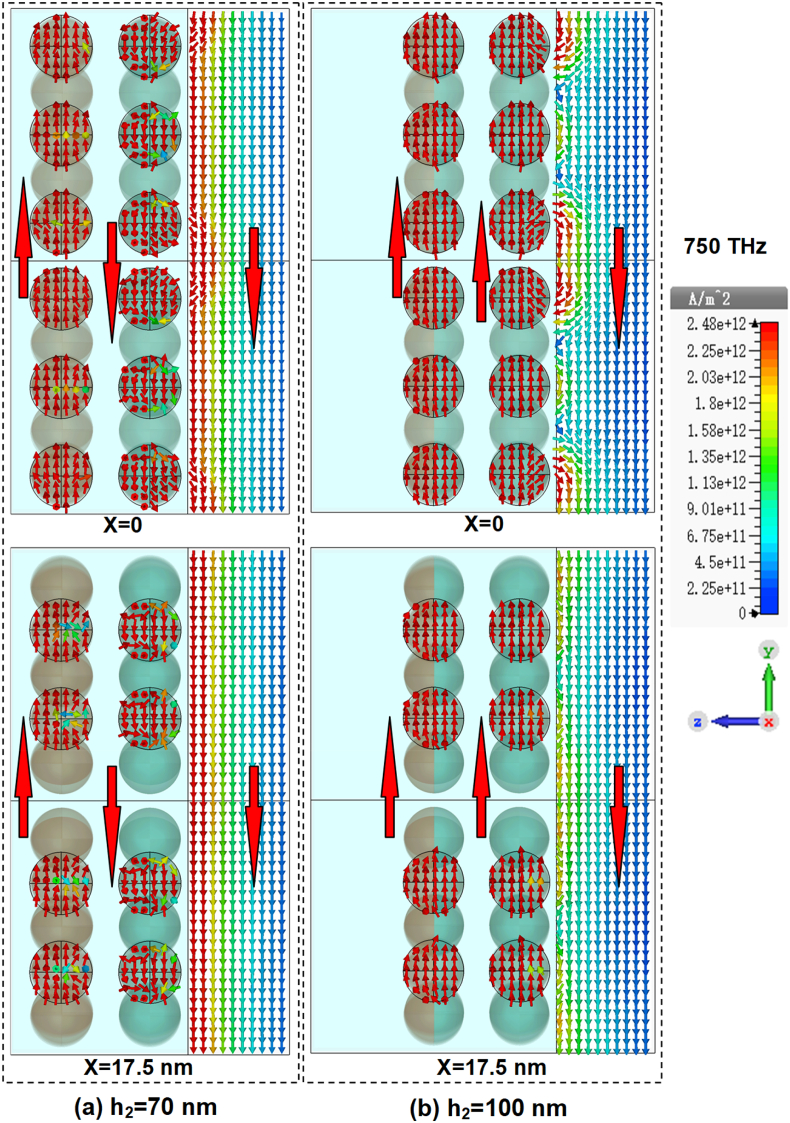
Distributions of the induced current density in Matrix (n = 2): Cu/Al + Al. (a) $h_{2}=70$ nm; (b) $h_{2}=100$ nm.

**Fig. 11:**
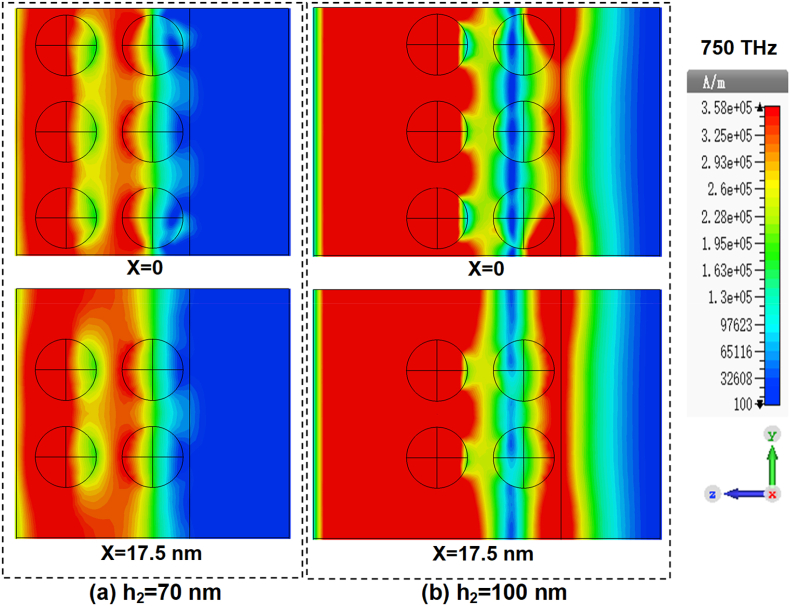
Distributions of the magnetic field in Matrix (n = 2): Cu/Al + Al. (a) $h_{2}=70$ nm; (b) $h_{2}=100$ nm.

To scrutinize the physical mechanism underlying the influence of matrix thickness on absorption, we plot the induced current density within the composites films for $h_{2}=70$ nm and $h_{2}=100$ nm in Fig. 10(a) and Fig. 10(b), respectively. Under the action of incident electromagnetic field, the induced currents are coming into being in alloy-particle sublayers and the metal layer. For the structure with $h_{2}=100$ nm, the induced currents in both alloy-particle sublayers are in the same direction but are in opposite direction with that in the base metal layer. The closed current loop forms strong magnetic resonances, as indicated by the magnetic field distributions in Fig. 11(a) and Fig. 11(b). It is these magnetic resonances that are responsible for the absorption in the composites. While for $h_{2}=70$ nm, the phases of the electric field interacting with both the alloy particles and the metal layer are changed. The induced current in inner alloy-particle sublayer then is in the same direction with that in base metal film and the magnetic resonance cannot form in between. Consequently, the overall magnetic resonances are weakened and the field power absorbed is decreased.

**Fig. 12:**
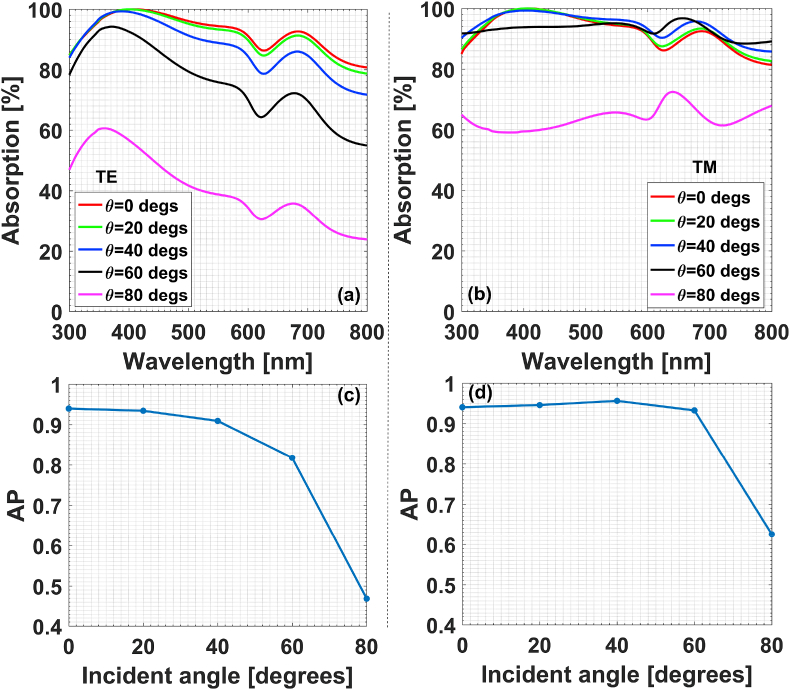
Effect of incidence obliquity on absorption characteristics of composites Matrix (n = 2):Cu/Al + Al for TE and TM polarization: (a, c) TE polarization; (b, d) TM polarization.

The dependence on incidence obliquity is discussed and listed in Fig. 12, in which θ=0 corresponds to the normal incidence. The structure configuration is the case in Fig. 9(b) with $g_{i}=35$ nm and $h_{2}=100$ nm. When the incidence angle is getting larger, the change of field-regulating characteristics for TE polarization is kind of monotonous, as shown in Fig. 12(a) and (c). When the incident angle is less than 60°, the value of AP is larger than 0.8, which means that over 80 percents of incident field power can be absorbed by the multilaminar composites. While for TM polarization, the absorption performance is even better and over 90 percents of incident field power can be absorbed even if the incident angle increases up to larger than 60°, as shown in Fig. 12(b) and (d). Going beyond, one can add more layers of alloy particles with complicated spatial distribution in matrix to tailor the field-regulating properties of multilaminar plasmonic films. Owing to space limitation, the results will not be presented here.

Last but not least, the fabrication method is also an important issue. With regard to the composites embedding metal nanoparticles, the most commonly used preparation method is magnetron sputtering. As for the composites embedding pure-metal particles combination, e.g., Au–Ag combination, one can use two independent sputtering targets in sputtering instrument to fabricate the material. While for the composites embedding alloy particles, e.g., Au/Ag alloy, the sputtering target simultaneously containing two metal components can be specifically made and used for fabrication. reference [25] can serve as a practical reference for experimental realization.

## 3 Conclusions

We have analyzed the field-regulating characteristics of alloy-based metamaterial composite films in 300–800 nm. Firstly, we proposed a petal-like microstructure analysis model for alloy particle and determined the extent of subdivision to be sixteen petals. Based on this model, we found the alloy plasmonic effect that Au/Ag alloy particles support a broader resonance band with higher averaged resonance intensity than pure silver or gold particles or the combination of both. As for the alloy-particle-based composites, there exists the combined action of inter- and intra-sublayer coupling interactions and the composites show saturation effect in its field-regulating characteristics. This means that embedding more alloy particles does not definitely guarantee the enhancement of field absorption. Taking into consideration of both the alloy plasmonic effect and the absorption saturation effect, we further explored the plasmonic field-regulating characteristics of alloy-based multilaminar films and finally demonstrated a broadband perfect absorber including a layer of Cu/Al alloy-particle-based composites and an aluminum base layer. In addition, a quality factor of absorption performance is purposely introduced to quantify the field-regulating characteristics. The value of AP of the achieved absorber reaches 0.94 throughout the range of 300–800 nm. The results will serve as a direct reference for designing alloy-based plasmonic metamaterials.

Although we tried our best to give as detailed an analysis as possible, the limitations still exist. In the process of modeling, for the sake of simplicity, we just assumed spherical shape for both pure metal and alloy particles. However, in almost all real cases, the particles can take any other shapes and the shape does have nonnegligible impact on its plasmonic field-regulating characteristics. Therefore, from a more macroscopic and comprehensive perspective, the results obtained can only be used as a semi-quantitative reference for possible applications. To get more accurate description on the plasmonic characteristics of alloy-based film, one has to transit from ideal model to real model. This will not only increase the difficulty of modeling, but also put forward even higher requirements for computing power. Even so, this will still be what we are determined to carry out in the near future.

## Author contribution statement

Yifan Kang: Conceived and designed the experiments; Performed the experiments; Wrote the paper.

Hongtao Yang & Chao Wang: Conceived and designed the experiments; Analyzed and interpreted the data.

Yongfeng Li: Analyzed and interpreted the data; Contributed reagents, materials, analysis tools or data.

Peng Xu: Contributed reagents, materials, analysis tools or data; Wrote the paper.

## Funding statement

Prof. Chao Wang was supported by 10.13039/501100017596Natural Science Basic Research Program of Shaanxi Province [2022JZ-36], 10.13039/501100001809National Natural Science Foundation of China [11675258].

## Data availability statement

Data will be made available on request.

## Declaration of interest's statement

The authors declare that they have no known competing financial interests or personal relationships that could have appeared to influence the work reported in this paper.
